# First in class monoclonal antibody potentiating human follicle stimulating hormone activity improves spermatogenesis in azoospermic rodent models

**DOI:** 10.3389/fendo.2025.1668945

**Published:** 2025-10-14

**Authors:** Elodie Kara, Jérémy Decourtye, Laurence Dupuy, Sophie Casteret, Philippe Bouchard, René Frydman, Marie-Christine Maurel

**Affiliations:** ^1^ Igyxos Biotherapeutics, Centre INRAE Val de Loire, Nouzilly, France; ^2^ Hôpital Foch, Service de Gynécologie Obstétrique, Suresnes, France

**Keywords:** antibody, fertility, spermatogenesis, follicle stimulating hormone, azoospermia

## Abstract

**Introduction:**

*In vitro* and *in vivo* in rodent models were used to study the effects of a human follicle-stimulating hormone (hFSH)-potentiating monoclonal antibody (IGX12) on hFSH bioactivity.

**Methods:**

Potentiation of recombinant hFSH (rhFSH)-, human luteinizing hormone/chorionic gonadotropin (hLH/hCG)-, or human thyroid stimulating hormone (TSH)-mediated cyclic adenosine monophosphate (cAMP) induction *in vitro* was performed using HEK 293 cells overexpressing either human FSH receptor (hFSH-R), human LH-receptor (hLH-R) or human thyroid stimulating hormone receptor (hTSH-R). The effect of rhFSH on ovarian weight and hCG on seminal vesicle weight was tested in immature female or male rats. Potentiation of spermatogenesis was examined in adult male rats with acquired azoospermia and *hpg* mice homozygotic for the hypogonadal mutation (Gnrh1*^hpg^*).

**Results:**

IGX12 dose-dependently potentiated rhFSH induction of cAMP production *in vitro* with an approximately 30% larger maximum response and increased ovarian weight by 1.8 fold *in vivo* versus rhFSH alone. A higher concentration of IGX12 (200 μg/ml) demonstrated a slight potentiating effect on hLH or hCG *in vitro* and non-statistically significantly increased seminal vesicle weight by 1.3 fold *in vivo*. No potentiating effect of IGX12 on hTSH was observed. *In vivo*, the addition of IGX12 to exogenous gonadotropins in the rat acquired azoospermia model increased testes weight by 1.8 fold and sperm counts by 1.5 fold compared with gonadotropins alone and was more effective than doubling the gonadotropin dose. In the mouse congenital azoospermia model, versus gonadotropins alone, addition of IGX12 significantly improved sperm counts in the testes by 3.5 fold and in the epididymides by 5.5 fold.

**Conclusion:**

IGX12 is a first-in-class humanized monoclonal antibody (mAb) that potentiated FSH activity *in vitro* and stimulated spermatogenesis more effectively than gonadotropins alone in congenital and acquired azoospermia rodent models, even when increasing the dose of gonadotropins had no additional effect. These *in vitro* and *in vivo* findings suggest that IGX12 could have promise as a future treatment option for men with azoospermia and oligozoospermia.

## 1 Introduction

Male factor infertility is an initial or contributing cause for approximately half of couples experiencing fertility issues (1). The causes vary, and generally relate to congenital, acquired, or idiopathic factors that impair spermatogenesis (1). The worldwide prevalence of male infertility is approximately 1 in 10 (2, 3). Treatments include both pharmacological and non-pharmacological approaches to improve sperm parameters. However, a recent meta-analysis concluded that the current evidence is poor and that further well-designed studies are needed (4).

Follicle-stimulating hormone (FSH) plays a major role in spermatogenesis, justifying its use in male infertility treatment. Currently, gonadotropins are used on-label in the treatment of hypogonadotropic hypogonadism (HH), a rare disease; for all others causes of male infertility, such as oligozoospermia, they are used off-label (5, 6). However, whatever the cause of infertility, gonadotropin treatments have limited efficacy (7, 8).

Against this background, we report early preclinical findings with a first-in-class humanized anti-FSH monoclonal antibody, IGX12, that potentiates FSH activity and stimulates spermatogenesis in azoospermic animal models, an initial step in developing a novel approach to the management of male infertility. This research program is based on previous results reflecting that during gonadotropin treatment to manipulate reproduction in livestock species, anti-gonadotropin antibody development correlates, in some females, with a 100% birth rate and even hyperprolificity due to potentiating antibodies (9, 10).

These findings prompted the design of a humanized IgG4 FSH potentiating monoclonal antibody, IGX12, and assessment of its effects on gonadotropin activity in a series of animal models. Here we report on the male studies, demonstrating the impact of IGX12 on FSH bioactivity *in vitro* and its ability to stimulate spermatogenesis in azoospermic models.

## 2 Materials and methods

### 2.1 Materials

All hormones were commercially available. Recombinant human FSH (rhFSH, Gonal-f^®^) and recombinant human chorionic gonadotropin (hCG, Ovitrelle^®^) were from Merck Serono (Darmstadt, Germany). Native human luteinizing hormone (hLH), hCG and human thyroid stimulating hormone (hTSH) were from Cell Sciences (Newburyport, MA, USA). Menopur^®^, a human menopausal gonadotropin (hMG), and Firmagon^®^ (Degarelix), a gonadotropin-releasing hormone (GnRH) antagonist, were from Ferring Pharmaceuticals (Saint Prex, Switzerland). Cell lines were purchased from Innoprot (Derio, Spain) unless otherwise specified.

### 2.2 Animals

All *in vivo* experiments on animals were performed at Unité Expérimentale de Physiologie Animale de l’Orfrasière (UEPAO, Nouzilly, France, https://doi.org/10.15454/1.5573896321728955E12). *In vivo* experiments were carried out according to EU guidelines (Directive 2010/63/EU) on the protection of animals used for scientific purposes, evaluated by the ethics committee on animal experimentation of Région Centre-Val de Loire (France), and validated by the French Ministry of Research. Immature and adult Wistar Han rats were ordered from Charles River Laboratories (Saint Germain Nuelles, France). Cryopreserved *hpg* murine embryos carrying the hypogonadal mutation (Gnrh1*^hpg^*) and characterized by an overall underdevelopment of the reproductive tract were ordered from The Jackson Laboratory (Bar Harbor, Maine, USA) and revitalized at Charles River Laboratories (L’Arbresle, France). These embryos were used to develop adult, male mice homozygous for the hypogonadal mutation. Rats and mice were weighed at the end of acclimatation period and assigned to experimental groups by ensuring that animals’ weights were equally distributed in each group and the mean group weight and error bars were equal. During treatment, to ensure blinding, syringes were prepared by separate people from those who administered the product. Syringes were marked to show which animal to treat, without any indication about the treatment group. The number of animals per group was calculated to have a statistical power of 80% with an alpha risk threshold of 0.05. Rats and mice were euthanized using an automated CO_2_ machine, which induced a gradual phase of sleep within 3 steps with a flow of 30L/min to reach 50% O_2_/50% CO_2_, and a subsequent CO_2_ saturation (100% CO_2_) according to a secure protocol (TEMSEGA, Saint-Médard-en-Jalles, France).

### 2.3 Development of IGX12, a humanized monoclonal antibody directed against rhFSH

The CF12 mAb, was first obtained by immunization of mice with rhFSH (27). The murine antibody was then humanized by complementarity determining region (CDR) grafting (11, 12). The selected humanized variant, IGX12, was expressed in a stable Chinese hamster ovary (CHO) cell line (Selexis, Plan-les-Ouates, Switzerland) and production was scaled up under good manufacturing practice (GMP) conditions (Merck Biodevelopment, Martillac, France) after Chemistry, Manufacturing, and Controls (CMC) optimization.

#### 2.3.1 Size exclusion chromatography analysis

SEC analysis was performed using a AKTA pure 25 M1 system (GE Healthcare Europe GmbH) with a Superdex™ 200 10/300 Increase GL column (GE Healthcare Europe GmbH, 28-9909-44). Two hundred µg of IGX12 were loaded onto the column and separated with a PBS mobile phase (150 mM NaCl, 50 mM KH_2_PO_4_/K_2_HPO_4_ pH 7.4) at 1 ml/min flow rate. Chromatogram was obtained by monitoring the absorbance at 280 nm.

#### 2.3.2 Surface plasmon resonance

Interaction between rhFSH and IGX12 was measured by SPR using a T200 Biacore™ Instrument (GE Healthcare). Experiments were performed at 25°C using 10 mM HEPES, 150 mM NaCl (Cytiva) pH 5.7 as running and diluting buffer. Anti-human IgG (Fc) (Cytiva, Wilmington, USA) antibodies were immobilized on the surface of a sensor chip CM5 (Cytiva) following the manufacturer’s recommendations. IGX12 was first captured on the functionalized surface (700 RU) then increasing concentrations of rhFSH were injected at 14.6 nM, 29.3 nM, 58.6 nM, 117.2 nM, 234.4 nM, 468.8 nM, 937.5 and 1875 nM for rhFSH for 10 min at 10 µL/min in multi-cycle kinetics (MCK) mode. The dissociation of the bound antigen from the mAb surface was measured for 6 min. A blank cycle was performed with running buffer injection instead of Gonal-f^®^ and was subtracted from each cycle with Gonal-f^®^. The dissociation constant K_D_ was determined from the corrected sensograms by a steady-state affinity analysis model with the Biacore T200™ evaluation software.

### 2.4 HEK 293 hFSH-R GloSensor cells

HEK 293 cells overexpressing both the human FSH receptor (hFSH-R) and the cyclic adenosine monophosphate (cAMP)-responsive biosensor GloSensor™ (Promega, Charbonnières-les-bains, France) were kindly provided by Eric Reiter (INRAE Val de Loire, Nouzilly, France). They were cultured at 37°C, 5% CO_2_, in a humidified atmosphere, in minimal essential medium (MEM) supplemented with 10% heat inactivated foetal bovine serum, 100 IU/ml penicillin, 100 µg/ml streptomycin, 0.4 µg/ml geneticin, and 0.4 µg/ml hygromycin.

Cells were plated and cultured overnight in white 96-well plates at a density of 80,000 cells per well. Prior to stimulation, cell culture media was removed and replaced by 100 µl MEM containing 4 µl of GloSensor cAMP reagent in which cells were incubated for 2 h at room temperature. Luminescence was recorded using a POLARstar Omega plate reader (BMG Labtech, Champigny-sur-Marne, France) before stimulation. Cells were then stimulated with 10 µl of increasing concentrations of rhFSH either alone or pre-incubated with a fixed dose of IGX12 (10 µg/ml), or with 10 µg/ml of IGX12 alone, prepared and incubated in polymerase chain reaction (PCR) mixing blocks at 37°C for 20–25 min. In other experiments, cells were stimulated with a mix of increasing concentrations of IGX12 and a fixed dose of rhFSH. Immediately after stimulation, luminescence was recorded over 60 min. The maximal cAMP level was extrapolated from the kinetic curves and fitted to generate dose/response curves.

### 2.5 HEK 293 cells overexpressing LH/CG receptor or hTSH receptor

HEK 293 cells overexpressing hLH-R were cultured at 37°C, 5% CO_2_, in a humidified atmosphere, in Dulbecco’s MEM (DMEM) supplemented with 10% heat inactivated fetal bovine serum and 10 µg/ml of puromycin. They were plated in white 96-well plates at a density of 80,000 cells per well and cultured overnight prior to stimulation. Cells were starved for 2 h in 25 µl phosphate-buffered saline (PBS) containing 1 mM HEPES at 37°C, 5% CO_2_. Either different dilutions of native hLH or hCG with a fixed concentration of IGX12 (10 µg/ml or 200 µg/ml), or serial dilutions of IGX12 with a fixed dose of hLH (2 nM) or hCG (1 nM) were pre-mixed in PCR mixing blocks and incubated at 37°C for 20–25 min. The mixes (20 µl) were then added to the cells and incubated for 90 min at 37°C, 5% CO_2_. The produced cAMP level was quantified using HitHunter cAMP assay for Biologics (Eurofins, Celle-L’Evescault, France).

HEK 293 cells overexpressing human TSH receptor were cultured at 37°C, 5% CO_2_, in a humidified atmosphere, in DMEM supplemented with 10% heat inactivated fetal bovine serum and 250 µg/ml of geneticin. Cells were plated in white 96-well plates at a density of 80,000 cells per well, and cultured overnight prior to stimulation. Cells were starved for 2 h in 25 µl PBS containing 1 mM HEPES at 37°C, 5% CO_2_. Either different dilutions of hTSH with a fixed dose of IGX12 (10 µg/ml), or serial dilutions of IGX12 with a fixed dose of hTSH close to its EC_20_ (4.66 ± 1.75 nM) were pre-mixed in PCR mixing blocks and incubated at 37°C for 20–25 min. The mixes (20 µl) were then added to the cells and incubated for 90 min at 37°C, 5% CO_2_. The produced cAMP level was quantified using HitHunter cAMP assay for Biologics.

### 2.6 Steelman and Pohley bioassay

Immature (27–28 day-old) female rats received 3.5 IU rhCG + 0.25 IU rhFSH or 3.5 IU rhCG + 0.25 IU rhFSH + IGX12, twice-daily by subcutaneous injection over 3 days (13). Animals were euthanized on Day 4, the ovaries were dissected, and ovary weight was normalized per 100 g of body weight.

### 2.7 Van hell bioassay

Immature (26–27 day-old) male rats received either 1.5 IU rhCG only or 1.5 IU rhCG + IGX12, once-daily by subcutaneous injection over 4 days (14). The animals were euthanized on Day 5, the seminal vesicles were dissected, and seminal weight was normalized per 100 g of body weight.

### 2.8 Adult rats treated with GnRH antagonist

Animals were weighed and injected with 2 mg/kg of a GnRH antagonist at D1 and 6-weeks later (D42) to maintain the inhibition of spermatogenesis. Rats were randomized to different treatments 7-weeks after the first injection (D49).

Two groups were treated with hormones only as follows: 2.5 IU/kg rhCG twice a week (Tuesday and Friday) + 25 IU/kg hMG 5 days a week (Monday to Friday) (HormonesX1), or 5 IU/kg rhCG twice a week+ 50 IU/kg hMG 5 days a week (HormonesX2). IGX12 was added on top of HormonesX1 treatment and injected subcutaneously 3 times a week (Monday, Wednesday and Friday) at 20 µg/kg of body weight (**Supplemental Figure 1A**). The control group received saline 5-days a week.

Animals were euthanized after 8-weeks of treatment. Testicles and epididymides were dissected and weighed.

### 2.9 Hypogonadal mice

Adult male mice, homozygous for the mutation (*hpg* -/-) were treated with either hormones only or hormones + IGX12. The first group of mice was treated with 1 IU/animal/injection of rhFSH 5-days a week. The second group was treated with 1 IU/animal/injection of rhFSH 5-days a week + IGX12 injected twice weekly at 20 µg/kg body weight. After 7-weeks of treatment, rhFSH was replaced by hMG at a dose of 25 IU/kg, and the treatment was prolonged for an additional 7-weeks (**Supplemental Figure 1B**).

Animals were euthanized after 14-weeks of treatment. Testes and epididymides were dissected and weighed.

### 2.10 Sperm count in the testes

The testes were ground in Ham’s F12 medium supplemented with 1% sodium pyruvate and 1% HEPES and sonicated. The number of spermatid heads was counted using a Malassez hemocytometer to assess testicular sperm reserve.

### 2.11 Sperm count in the epididymis

Epididymides were cut into thin sections and incubated for at least 4 h at room temperature in Ham’s F12 medium supplemented with 1% sodium pyruvate and 1% HEPES. The preparations were recovered, filtered, and the spermatozoa were counted using a Malassez hemocytometer.

### 2.12 Flow cytometry

Sample analyses were performed by the flow cytometry platform of Infectious Diseases and Public Health department of INRAE Centre Val de Loire (UMR ISP 1282–37380 Nouzilly, France). Mice testes and epididymides samples were analyzed using a MoFlo AstriosEQ high speed cell sorter (Beckman Coulter Inc, Brea, CA, USA) and Summit 6.3 software (Beckman Coulter Inc). Analysis was triggered with a blue laser (488 nm), debris was eliminated based on morphological criteria using forward scatter (FSC) *vs* side scatter (SSC). Hoechst 33342 was used to measure DNA content; it was excited using a violet laser (405 nm) and the blue fluorescence emitted was measured trough a 448/59 nm band pass filter. Different populations with increasing Hoechst fluorescence, representative of haploid (spermatids in testis and spermatozoa in epididymis), diploid and tetraploid (spermatocytes I) cells were detected. To improve the quantification of the different populations we analyzed the whole prepared sample. Sample analysis was performed using Kaluza Analysis 2.1 software (Beckman Coulter Inc.).

### 2.13 Quantification of IGX12 in adult rat plasma

The method was based on a sandwich enzyme immuno-assay. A volume of 100 µl/well of streptavidin (Sigma-Aldrich, Merck Millipore, Darmstadt, Germany) at 1 µg/ml in carbonate-bicarbonate buffer 0.1 M pH 9.6, was coated into MaxiSorp 96-well plate (NUNC, Dustcher, Brumath, France), incubated 1 h at 37°C, and overnight at +4°C. After a washing step with PBS/Tween 0.1%, 100 µl/well of PBS/Tween 0.1%/BSA 1% was added and incubated 1 h at 37°C. Rabbit anti-human IgG biotinylated Antibody 1 µg/mL (Invitrogen, Saint Aubin, France) was added (100 µl/well) and incubated 1 h at 37°C. After a further washing step, standards and samples were transferred (100 µl/well) in duplicate. Standard curve calibrators were prepared with IGX12 spiked in 1/100 pooled blank rat plasmas. IGX12 concentrations were set between 0.31 ng/ml and 50 ng/ml. Plasma samples were diluted 100 times in PBS/Tween 0.1%/BSA 0.2%. The plate was washed and 100 µl/well of peroxidase detection mouse anti-human IgG4 pFc’ antibody (Abcam, Rozenburg, Netherlands) diluted 1/2500 was added and incubated 1 h at 37°C. After a final washing step, TMB substrate solution was added (100 µl/well). The color reaction was stopped by the addition of a sulphuric acid 1 M solution and the color intensity measured at 450 nm. IGX12 concentrations from sample plasmas were extrapolated from the calibration curve.

### 2.14 Intratesticular testosterone in hpg mice

After testicular sperm reserve assessment, the sample is used to quantify the intratesticular testosterone levels using a commercially available kit from Cayman (Interchim, Montluçon, France).

### 2.15 Statistical analysis

Statistical analysis was performed using Prism software (GraphPad Software, Inc., San Diego, CA, USA). Quantitative variables were compared by using a Mann-Whitney test (male immature rats and *hpg* mice), or a Kruskal-Wallis test with a Dunn’s post-test (female immature rats and adult rats). Differences were considered significant when p<0.05.

## 3 Results

### 3.1 Characterization of IGX12

**Figure 1:**
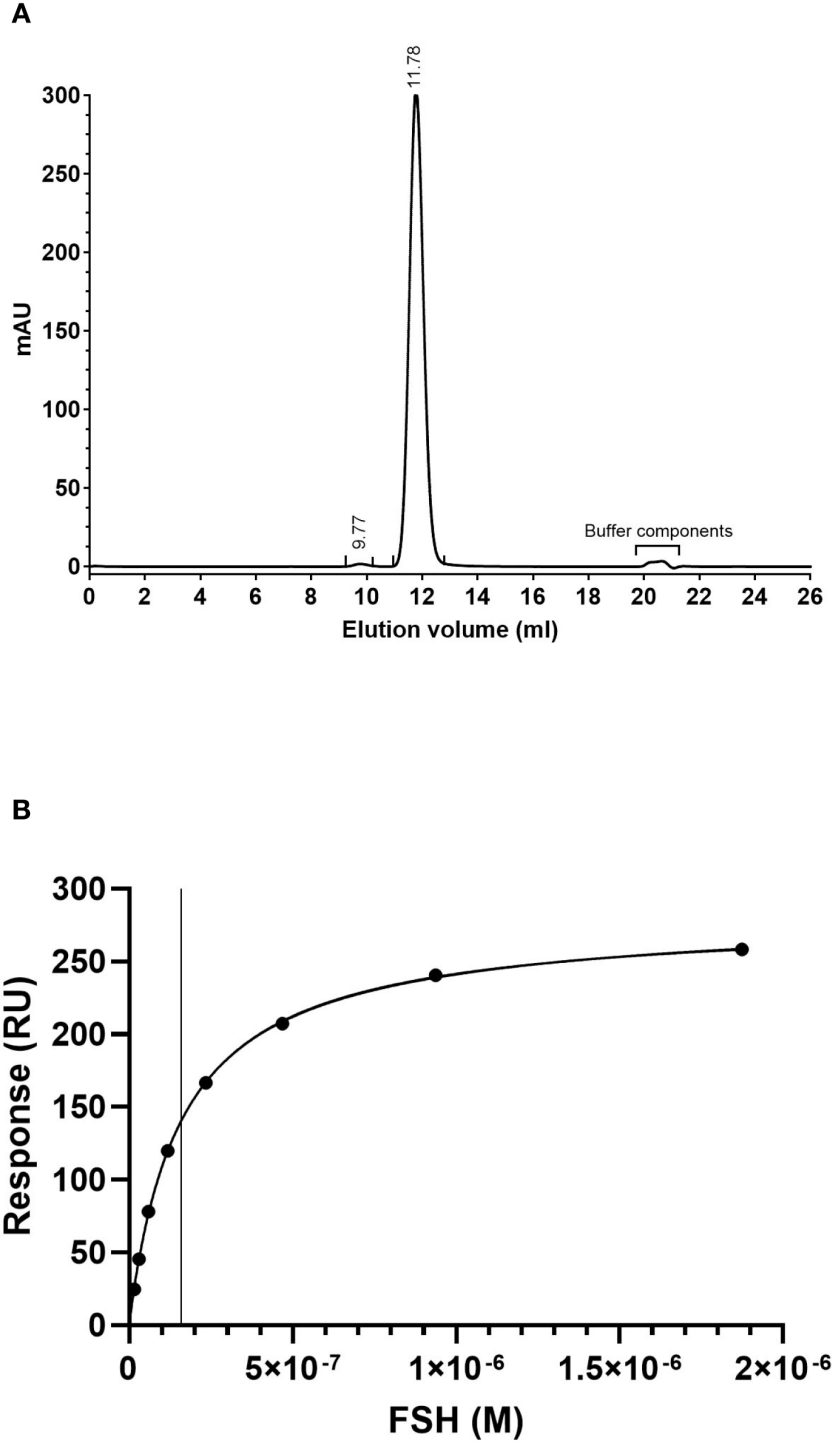
**(A)** Size exclusion chromatography profile of IGX12. Two hundred µg of IGX12 were analyzed using Superdex™ 200 10/300 Increase GL column. **(B)** SPR analysis of rhFSH binding on IGX12.

Size exclusion chromatography analysis of IGX12 at 280 nm shows a profile of a typical non-aggregated IgG mAb with a main monomer peak representing 99.6% of the mAb suspension and few aggregates at 9.77 ml retention volume. Peaks between 20−21 ml are sample formulation buffer components (**Figure 1A**).

The dissociation constant for the interaction between rhFSH and IGX12 was 1.60 x 10–^7^ M (**Figure 1B**).

### 3.2 *In vitro* and *in vivo* effect of IGX12 on rhFSH

**Figure 2:**
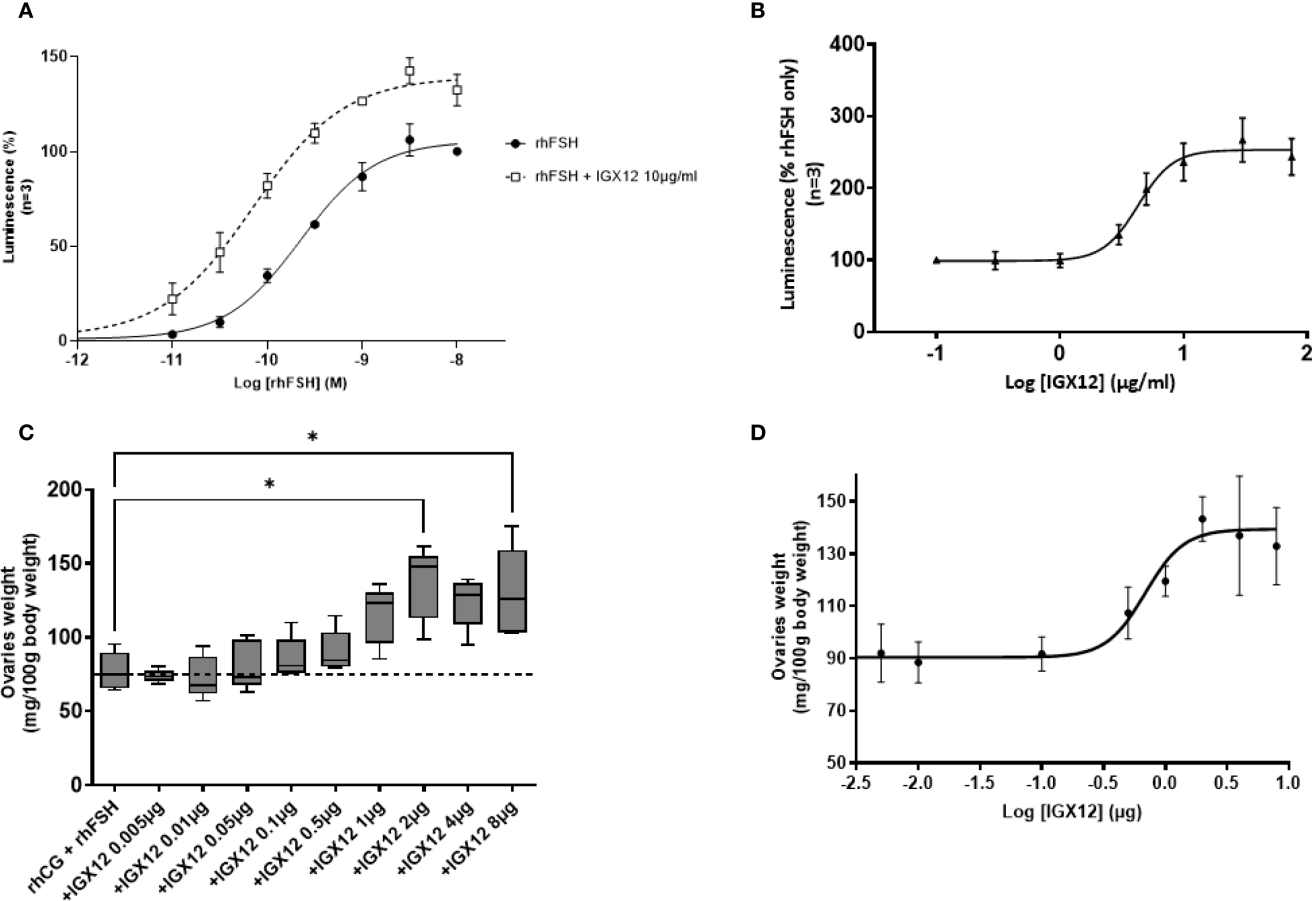
*In vitro* and *in vivo* effect of IGX12 on rhFSH. **(A)** *In vitro* effect of 10 µg/ml of IGX12 on increasing concentrations of rhFSH. Mean ± SEM of 3 experiments. **(B)** *In vitro* effect of increasing concentrations of IGX12 on EC_20_ of rhFSH. Mean ± SEM of 3 experiments **(C)** Effect of increasing doses of IGX12 on 3.5 IU rhCG + 0.25 IU rhFSH in Steelman and Pohley bioassay. n=5 animals per group. Results were expressed as median with 10–90 percentile. **(D)** Dose response curve of IGX12’s *in vivo* effect on rhFSH extrapolated from values shown in **(C)** (*p < 0.05).

HEK 293 cells overexpressing hFSH-R were stimulated with increasing doses of rhFSH, and cAMP production was measured. When the different doses of rhFSH were pre-mixed with a fixed dose of IGX12 (10 µg/ml) before addition to cells, the dose/response curve was shifted, reducing the rhFSH concentration required to provide a half-maximal response (EC_50_, from 311 ± 75.6 pM to 111 ± 2.7 pM for rhFSH and rhFSH+IGX12 respectively). The maximum response (Emax) of rhFSH-induced cAMP was increased by ~30% (**Figure 2A**). As a control, cells stimulated with IGX12 only did not show any cAMP production, demonstrating that IGX12 acts via rhFSH and did not stimulate hFSH-R on its own in the absence of rhFSH (data not shown). A dose of rhFSH (50 pM) close to its EC_20_ (66.6 ± 25.1 pM) was used to generate a dose/response curve with increasing concentrations of IGX12, from 0.1 µg/ml to 200 µg/ml. IGX12 showed a dose dependent effect on rhFSH, with an EC_50_ of 4.1 ± 1.0 µg/ml (**Figure 2B**).

The *in vivo* effect of IGX12 on rhFSH was tested in the Steelman and Pohley bioassay. IGX12’s effect on ovaries was dose dependent, with an EC_50_ of 0.73 µg/injection. Maximal response was observed at 2 µg/injection, with ovary weight being 1.8 fold higher in the presence of IGX12 versus rhCG+rhFSH only (*p<0.05; **Figures 2C, D**).

### 3.3 *In vitro* and *in vivo* effect of IGX12 on hLH/hCG

**Figure 3:**
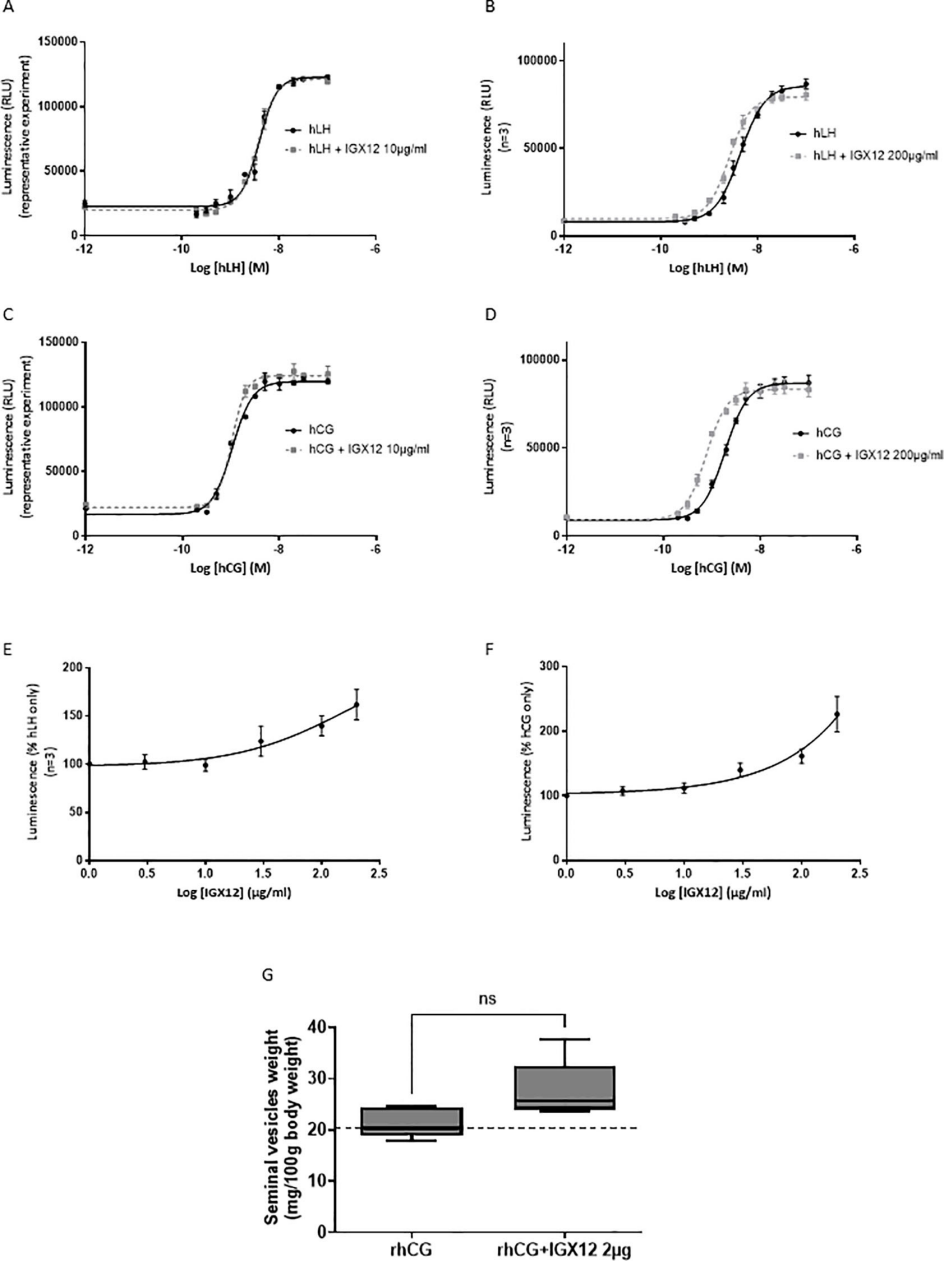
*In vitro* and *in vivo* effect of IGX12 on hLH and hCG. *In vitro* effect of IGX12 on increasing concentrations of hLH at 10 µg/ml **(A)** or 200 µg/ml **(B)**, and on hCG at 10 µg/ml **(C)** or 200 µg/ml **(D)**. *In vitro* effect of increasing concentrations of IGX12 on EC_20_ of hLH **(E)** or hCG **(F)**. **(A, C)** are representative experiments done in duplicate; **(B, D, E)** and **(F)** are mean ± SEM of 3 experiments. **(G)** Effect of 2 µg/injection of IGX12 on 1.5 IU hCG in Van Hell bioassay. n=5 animals per group. Results were expressed as median with 10–90 percentile (ns, non-significant).

HEK 293 cells expressing hLH-R were stimulated with increasing doses of either hLH or hCG. The HEK 293 hLH-R cells produced cAMP under stimulation, with an EC_50_ of 4.67 ± 1.29 nM for hLH and 1.89 ± 0.41 nM for hCG. The addition of IGX12 at 10µg/ml to different doses of hLH or hCG had no effect on both hormones (**Figures 3A, C**), whereas IGX12 showed a slight potentiating effect at 200 µg/ml (**Figures 3B, D**). The dose/response curve was shifted, exhibiting an EC_50_ of 2.7 ± 0.4 nM for hLH and 0.77 ± 0.06 nM for hCG, and Emax was unchanged. A dose of hLH (2 nM) and of hCG (1 nM) close to their respective EC_20_ was selected to generate a dose/response curve of IGX12 with concentrations ranging from 0.1 µg/ml to 200 µg/ml (**Figures 3E, F**). We were unable to generate a saturating dose response curve and extrapolate an EC_50_ for IGX12 activity for either hLH and hCG. Together, these results showed a slight effect of IGX12 *in vitro* on hLH and hCG at higher concentrations.

IGX12 effect on rhCG was tested *in vivo* in the Van Hell Seminal vesicles weight gain assay. IGX12 was tested at 2 µg/injection, which produced the maximum effect on FSH bioassay *in vivo*. The addition of IGX12 to rhCG improved the weight of seminal vesicles by 1.3 fold versus rhCG only. The effect of IGX12 on rhCG was lower than that observed with rhFSH in the Steelman and Pohley bioassay and was non statistically significant (**Figure 3G**).

**Figure 4:**
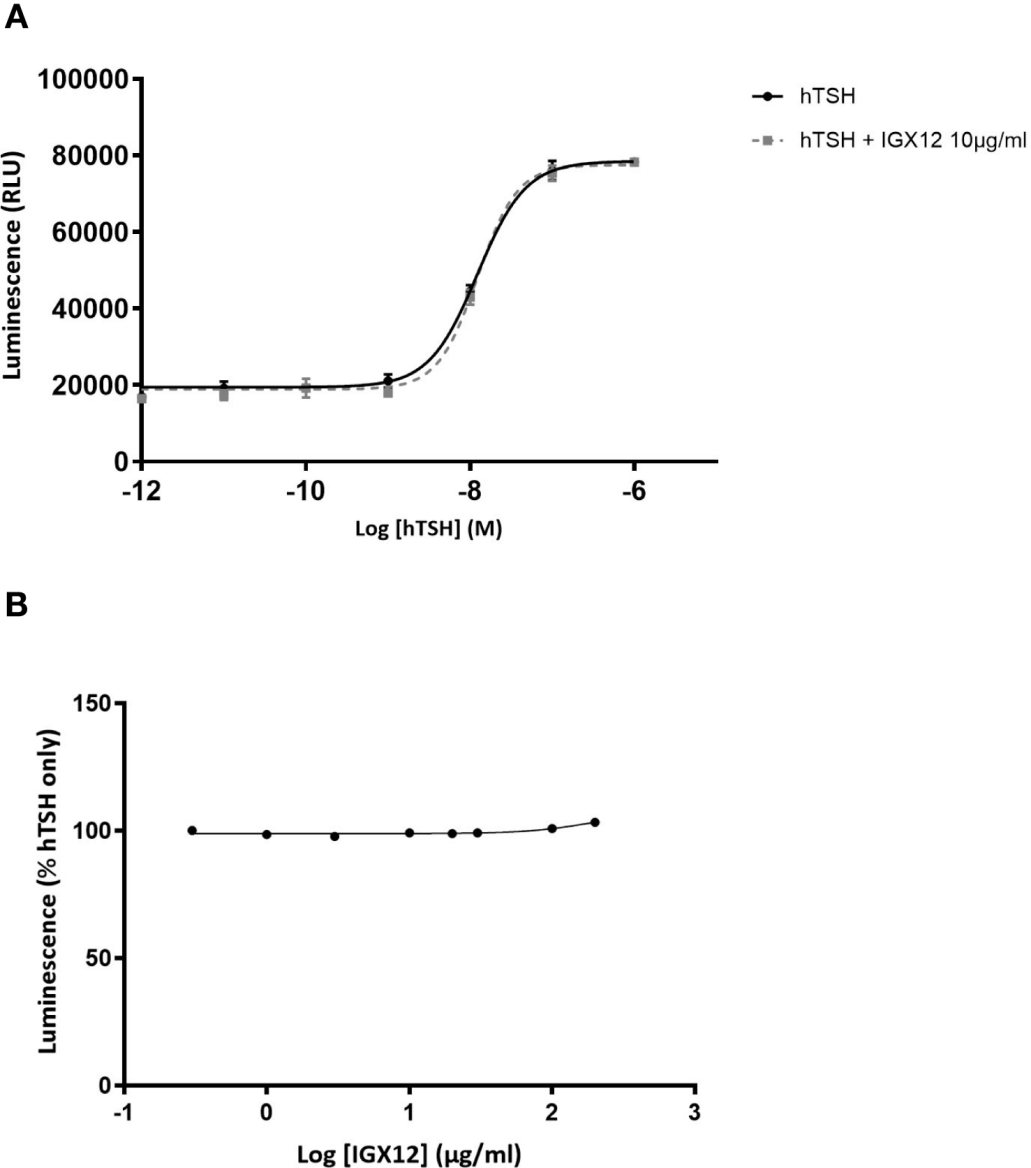
*In vitro* effect of IGX12 on hTSH on HEK 293 cells expressing hTSH-R. Cells were stimulated with increasing doses of hTSH, with or without 10 µg/ml of IGX12 **(A)**, or with increasing concentrations of IGX12 in the presence of 0.6 nM hTSH **(B)**.

There was no effect of IGX12 on hTSH, either in the dose/response of hTSH or dose/response of IGX12 on EC_20_ of hTSH (**Figure 4**).

### 3.4 *In vivo* effect of IGX12 in a rat acquired azoospermia model

**Figure 5:**
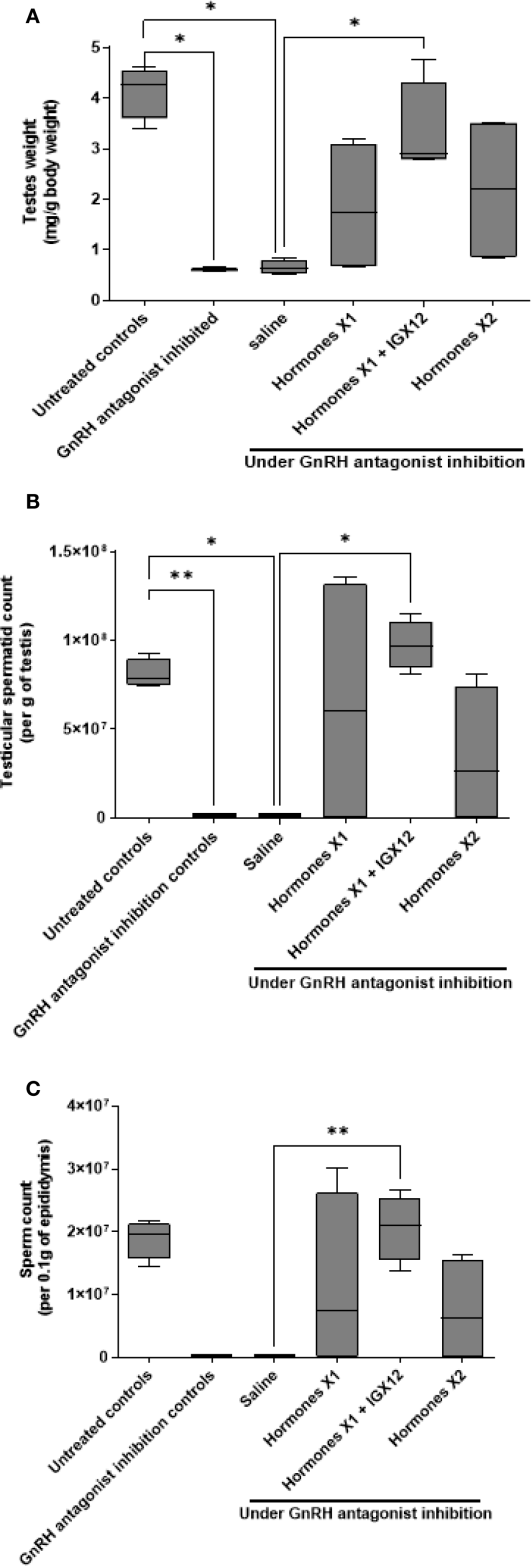
Effect of IGX12 in azoospermic rats. Effect of the addition of IGX12 on top of a hormonal treatment (rhCG+hMG) on testes weight **(A)**, testicular sperm count **(B)** and sperm count in epididymides **(C)**. n=4 rats per group. Results were expressed as median with 10–90 percentile (*p < 0.05 and **p<0.01).

The efficacy of IGX12 to restore spermatogenesis in an azoospermic adult rat model was tested. Rats were treated with a long-acting GnRH antagonist, resulting in HH. Seven-weeks after the first injection of the GnRH antagonist, once the testes weight of rats was significantly reduced (**Figure 5A**, *p<0.05), 8-week treatment with either saline, HormonesX1, HormonesX1 + 20 µg/kg IGX12, or HormonesX2 was started. The saline group had significantly smaller testes compared to untreated wild-type controls (**Figure 5A**, 0.65 ± 0.08 mg of testes weight/g of body weight, vs 4.13 ± 0.3 mg/g of body weight *p<0.05). Treatment with gonadotropins at the lower dose (HormonesX1), increased testes weight to 1.84 ± 0.78 mg/g of body weight. Doubling the gonadotropin dose (HormonesX2) did not improve the weight of testes (2.19 ± 0.88 mg/g of body weight). However, the addition of IGX12 to HormonesX1 treatment increased the testes weight by 1.8 fold compared to HormonesX1 alone (3.33 ± 0.55 mg/g of body weight), and was significantly different compared to saline (*p<0.05).

Rats treated for 7-weeks with GnRH antagonist only had no spermatids in the testes. After 8-weeks of saline treatment, only 1 of the 4 animals had a very low number of spermatids in the testes (mean 22.7 x 10^3^ ± 26.2 x 10^3^ per g of testes), and a very low sperm count in the epididymides, (mean 1.23 x 10^3^ ± 1.2 x 10^3^ per 0.1g of epididymis, **Figures 5B, C**). Eight-week gonadotropin treatment started spermatogenesis, but doubling the dose of hormones did not improve the sperm count in the testes (**Figure 5B**, 64 x 10^6^ ± 42.8 x 10^6^ and 33.5 x 10^6^ ± 23 x 10^6^ respectively for HormonesX1 and HormonesX2), whereas adding IGX12 on top of HormonesX1 significantly improved the spermatid count in the testes by 1.5 fold (**Figure 5B**, 97.3 x 10^6^ ± 8 x 10^6^, *p<0.05 compared to saline). Similarly, sperm counts in the epididymides with HormonesX1 or HormonesX2 were not significantly different (**Figure 5C**, 11.2 x 10^6^ ± 7.1 x 10^6^ and 7.3 x 10^6^ ± 4.2 x 10^6^ respectively) but were increased by 1.84 fold when IGX12 was added to HormonesX1 (**Figure 5C**, 20.6 x 10^6^ ± 2.7 x 10^6^, **p<0.01 when compared to saline). Spermatid counts in the testes in group treated with HormonesX1+IGX12 were at the same level as untreated control wild-type animals that were not treated with GnRH antagonist (**Figure 5B**). In conclusion, when IGX12 was added on top of hormonal treatment, spermatogenesis was fully restored in azoospermic rats within one spermatogenetic cycle only, whereas high doses of gonadotropins failed to do so. All together, these data demonstrate the potentiating effect of IGX12 on FSH *in vivo* and its ability to restore spermatogenesis more efficiently than gonadotropins only.

Finally, IGX12 concentration in the plasma of 2 adult rats was measured in plasma from blood collected at sacrifice. We detected on average ~2 µg/ml of IGX12 in the plasma at the end of the 8-week treatment, suggesting a concentration under half of the *in vitro* EC_50_ measured in HEK 293 hFSH-R GloSensor cells (4.1 ± 1.0 µg/ml).

### 3.5 *In vivo* effect of IGX12 in a congenitally azoospermic mice model (hpg mice)

**Figure 6:**
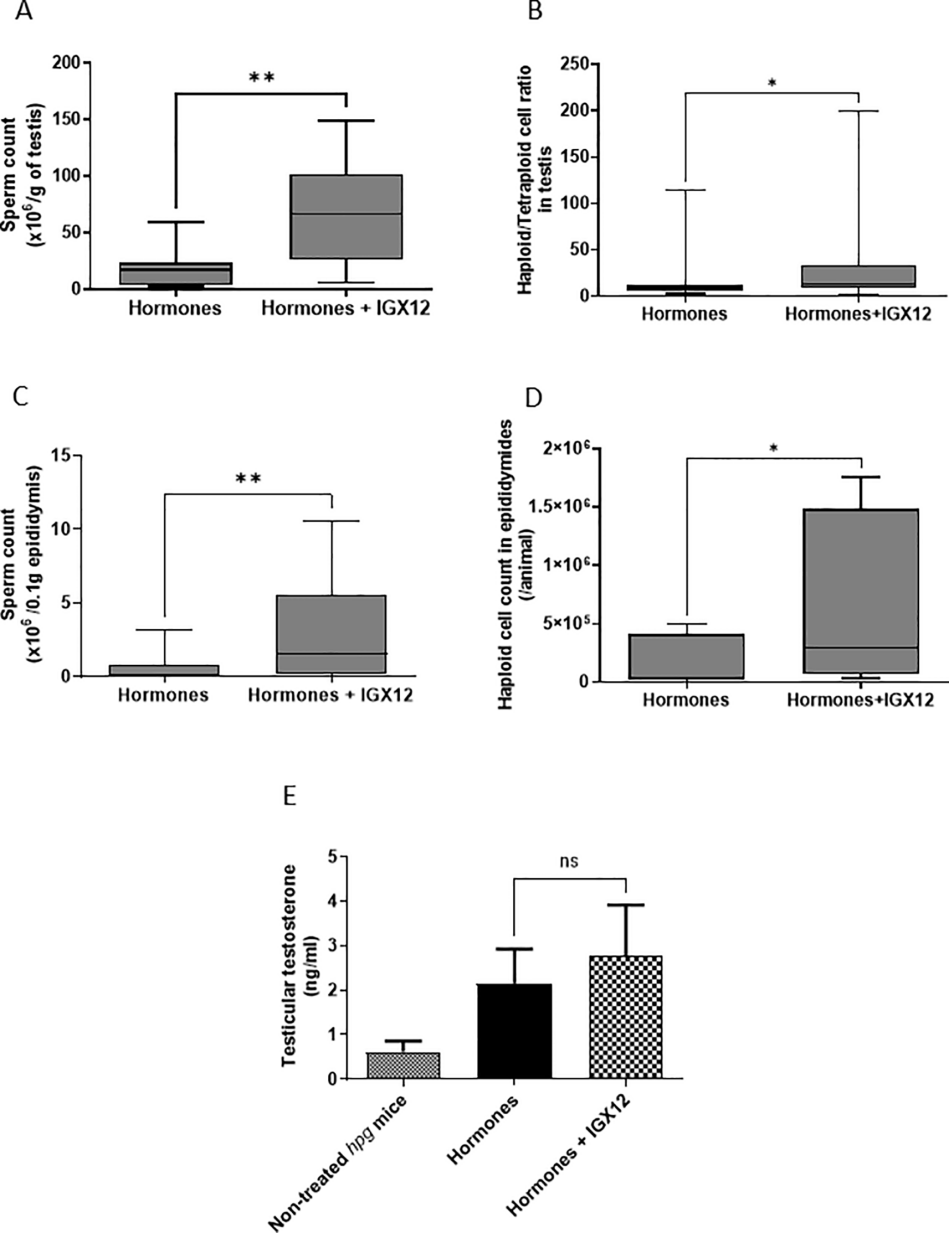
Effect of IGX12 in *hpg* mice. Testicular sperm count **(A)**, haploid/tetraploid cell ratio in testes **(B)**, sperm count in epididymides **(C)**, haploid cell count in epididymides **(D)** and intratesticular testosterone level **(E)** after treatment with either rhFSH and then hMG, or rhFSH+IGX12 and then hMG+IGX12. n=8 mice per group. Results were expressed as median with 10–90 percentile (*p<0.05 and **p<0.01) ns stands for non-significant.

*Hpg* mice were initially injected with rhFSH with or without IGX12 over 7-weeks. Then, rhFSH was replaced by hMG and the treatment was continued for an additional 7-weeks, with or without IGX12. At the beginning of treatment, *hpg* mice had no spermatids in the testis or spermatozoa in the epididymis. After 14-weeks of treatment, spermatids appeared in the testes, as well as spermatozoa in the epididymis. When IGX12 was added to the treatment, sperm counts in the testes were significantly increased from 18.7 x 10^6^ ± 7.5 x 10^6^ to 67.0 x 10^6^ ± 18.8 x 10^6^ spermatids/g of testis (**Figure 6A**, ** p<0.01). The ratio of haploid cell/tetraploid cell in each testes was also significantly increased when treated with IGX12, demonstrating an increase in haploid cell count (**Figure 6B**, 18.02 ± 9.06 versus 28.32 ± 11.06 in groups treated with hormones or hormones+IGX12 respectively, *p<0.05). When IGX12 was added to the treatment, sperm counts in the epididymis significantly increased from 0.6 x 10^6^ ± 0.4 x 10^6^ to 3.1 x 10^6^ ± 1.2 x 10^6^ spermatozoa/0.1 g of epididymis (**Figure 6C**, ** p<0.01). Haploid cell counts in the epididymis was also significantly increased from 1.6 x 10^5^ ± 0.8 x 10^5^ to 6.6 x 10^5^ ± 2.4 x 10^5^ when IGX12 was added to the treatment (**Figure 6D**, * p<0.05). Together, these data indicate that IGX12 significantly improved spermatogenesis stimulation in congenitally hypogonadal mice, when added to hormonal treatment.

Intratesticular testosterone levels were also assessed in treated *hpg* mice (**Figure 6E**). Similarly to what was observed in immature rats seminal vesicles weight gain bioassay, testosterone level tended to be higher in the presence of IGX12, without any statistical significance, demonstrating that, when used at an efficacious dose on FSH, IGX12 effect on LH/CG bioactivity present in hMG is limited.

## 4 Discussion

We report here on a potentiating humanized mAb, IGX12, that dose-dependently potentiates FSH activity both *in vitro* by increasing cAMP production by HEK 293 cells overexpressing hFSH-R and *in vivo* by increasing ovarian weight when administered with rhCG+rhFSH. In contrast, IGX12 showed only a slight potentiating effect on hLH- or hCG-induced cAMP production in HEK 293 cells expressing hLH-R *in vitro*. *In vivo*, IGX12 showed a non-statistically significant effect on rhCG in the seminal vesicles weight gain assay. No potentiating effect on hTSH was observed *in vitro*, demonstrating a high specificity of IGX12 for hFSH. The mechanism of action of IGX12 is under investigation to better understand its effect on FSH affinity to its receptor and receptor internalization. We speculate that the potentiating effect probably involves the beta subunit that confers the specificity to glycoprotein hormones, as TSH is not potentiated by IGX12 and its effect on LH/CG is not significant.

*In vivo*, even though on a limited number of animals, the addition of IGX12 to exogenous gonadotropins in the rat acquired azoospermia model significantly increased testes weight and sperm counts compared with gonadotropins alone. Similar findings were observed in the mouse congenital azoospermia model, in which the addition of IGX12 to sequential treatment with rhFSH followed by hMG for 7-weeks each, significantly improved sperm counts and increased the proportion of haploid to tetraploid cells, indicating higher proportions of spermatids and spermatozoa and demonstrating a higher level of spermatogenesis. We did not observe any adverse events in any animals in any of these experiments.

The treatments used in the rat and mouse azoospermia models reflect typical medical practice for men with HH (15–17) that utilizes exogenous gonadotropin treatment to achieve spermatogenesis (15, 16). The rat model, in which animals were treated with rhCG+hMG, reflects typical treatment approaches for postpubertal HH onset (16). The mouse model, in which initial rhFSH treatment was followed by hMG treatment, reflects treatment approaches in men with prepubertal HH onset (16). In such cases, FSH pretreatment may be used to induce Sertoli cells and spermatogonia proliferation, thereby increasing testes volume, which is then followed by gonadotropin treatment to achieve spermatogenesis (16).

While different treatment approaches have been developed, in general, the efficacy of gonadotropin treatment for HH is limited with only 50-80% of treated patients achieving spermatogenesis targets even after extended treatment periods. For example, after treatment with FSH and hCG, it was reported that 50% of men with HH and azoospermia at baseline had a sperm concentration ≥ 1.5 x 10^6^/ml at 9 months (18), 64.3% at 12 months (19) and from 63.2% (20) to 80% (21) at 18 months.

In the rat model, doubling the dose of gonadotropins did not improve sperm counts compared with the initial gonadotropin dose. In contrast, the addition of IGX12 to the initial dose significantly improved sperm counts. These data support the findings from clinical studies reviewed above, which show that the efficacy of gonadotropin treatment is limited in achieving sperm concentrations compatible with pregnancy. Thus, simply increasing the dose of gonadotropins may not always be associated with improvements in fertility outcomes (22–24). In contrast, adding IGX12 to gonadotropins in the rat model improved outcomes that increasing the gonadotropin dose was unable to achieve. Therefore, these findings suggest that IGX12 has the potential to improve current spermatogenesis treatment and consequently to reduce the duration of treatment required to reach sufficient sperm concentration for fertility outcomes.

IGX12 should be relevant for the treatment of a range of male fertility disorders. While the evidence supporting FSH treatment for HH is well established; data supporting empiric, off-label use of FSH in male idiopathic infertility is less clear cut (25). Real-world evidence suggests that FSH is considered a therapeutic option by clinicians in cases of male idiopathic infertility with impaired sperm parameters (7, 25). It is estimated, however, that 10 men would need to be treated to enable one spontaneous pregnancy, while 18 men would need to be treated to achieve one pregnancy using assisted-reproduction technology (26). These high number needed to treat values suggest that there is an unmet need for treatment optimization in idiopathic male infertility. Thus, IGX12, which potentiates FSH activity, could be a promising new therapeutic approach to explore in men with idiopathic infertility including oligozoospermia, who represent a sizable proportion of patients with infertility.

The clinical consequences of FSH hyperstimulation are different in men and women. In women, the main side effect of a high level of FSH activity is the ovarian hyperstimulation syndrome (OHSS) (14). However, in men, even though the amount of FSH required is much higher (2000–5000 IU for 1 cycle in women versus 10 000-30–000 IU in men), hyperstimulation does not occur and no serious adverse events or manifestations of FSH overdose were ever reported as a consequence of FSH replacement treatment in males. Additionally, FSH-producing tumours have no other effects than increasing testis size (14–16). It should be noted that IGX12 will not be used in cases of cancers that produce FSH.

In conclusion, IGX12 is a first-in-class humanized mAb, as to our knowledge it is the only therapeutic mAb that is capable of potentiating FSH activity. IGX12 was able to stimulate spermatogenesis more effectively than gonadotropins alone in congenital and acquired azoospermia rodent models, even when increasing the dose of gonadotropins had no additional effect in the rat model. Even though they were obtained in rodent models, these *in vitro* and *in vivo* findings suggest that IGX12 could be a promising future option for the treatment of men with azoospermia and oligozoospermia. Prior to that, its safety, tolerability and efficacy need to be confirmed in clinical trials.

## Data Availability

The datasets presented in this article are not readily available because they include proprietary preclinical data. Requests to access the datasets should be directed to elodie.kara@igyxos.com.
