# Supplementary material for: First in class monoclonal antibody potentiating human follicle stimulating hormone activity improves spermatogenesis in azoospermic rodent models
Source: Front Endocrinol (Lausanne). 2025 Oct 14;16:1668945. doi: 10.3389/fendo.2025.1668945 (PMC12558798; doi:10.3389/fendo.2025.1668945)
Supplement: Supplementary file 1 [file DataSheet1.docx]

**Supplemental Data**

Supplemental Figure 1: Diagram of treatment of adult rats (A) and *hpg* mice (B)


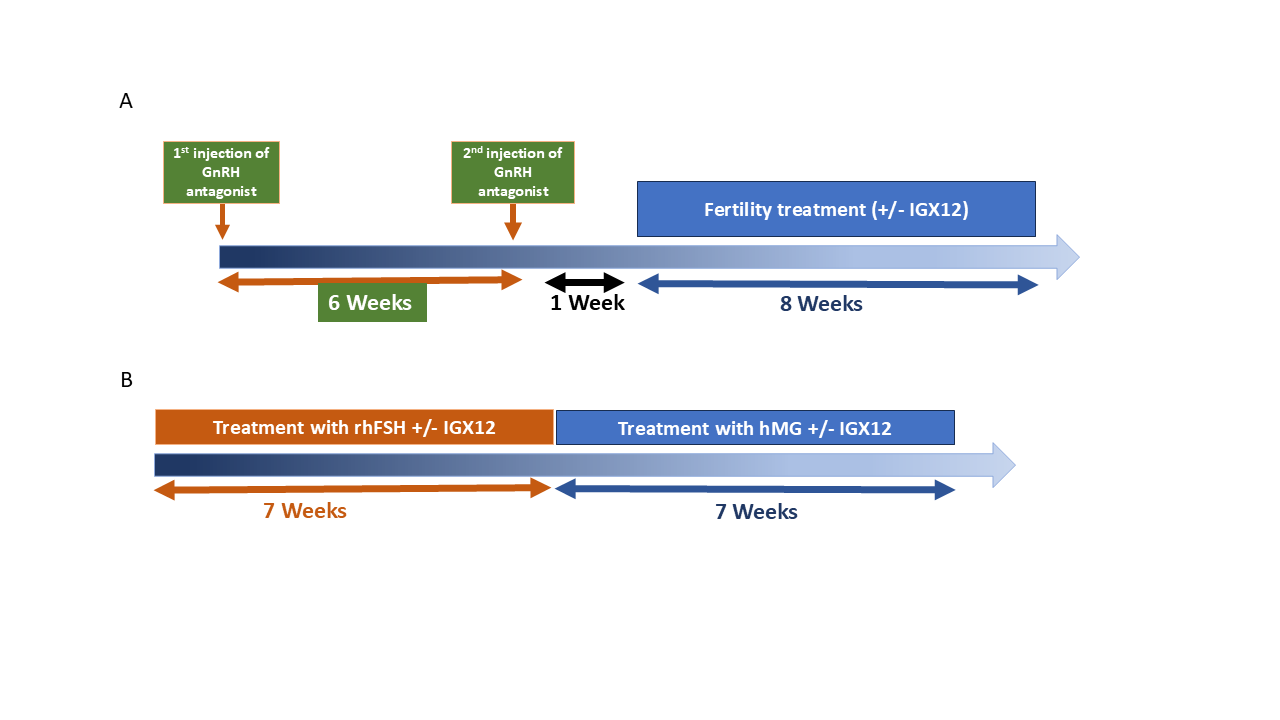


GnRH, gonadotropin-releasing hormone; hFSH, human follicle-stimulating hormone;
hMG, human menopausal gonadotropin; IGX12, hFSH-potentiating monoclonal antibody; rhFSH, recombinant hFSH
